# Synergetic Action of Forskolin and Mevastatin Induce Normalization of Lipids Profile in Dyslipidemic Rats through Adenosine Monophosphate Kinase Upregulation

**DOI:** 10.1155/2021/6687551

**Published:** 2021-05-19

**Authors:** Aaser M. Abdelazim, Tamer Ahmed Ismail, Mosleh M. Abumaghaid, Islam M. Saadaldin

**Affiliations:** ^1^Department of Basic Medical Sciences, College of Applied Medical Sciences, University of Bisha, Bisha, Saudi Arabia; ^2^Department of Biochemistry, Faculty of Vet. Medicine, Zagazig University, Zagazig, Egypt; ^3^Department of Clinical Laboratory Sciences, Turabah University College, Taif University, P.O. Box 11099, Taif 21944, Saudi Arabia; ^4^Department of Laboratory Medical Sciences, College of Applied Medical Sciences, University of Bisha, Bisha, Saudi Arabia; ^5^Department of Physiology, Faculty of Veterinary Medicine, Zagazig University, Zagazig, Egypt

## Abstract

In the present study, we examined the synergetic effect of forskolin and mevastatin administration on lipid profile and lipid metabolism in omental adipose tissue in dyslipidemic rats. The study was conducted on forty male albino rats. The rats were randomly classified into four main groups of ten animals in each group as follows: *group A*, served as control nontreated; *group B*, rats that received Triton WR 1339 (500 mg/kg); *group C*, rats that received Triton WR 1339 with forskolin (100% FSK extract 0.5 mg/kg/day) for four weeks; and *group D*, dyslipidemic rats received both mevastatin and forskolin. At the end of the experimental period, blood and omental adipose tissue samples were collected, preserved, and used for biochemical determination of lipid profile and mRNA expression profile of adenylate cyclase (AC), hormone-sensitive lipase, respectively (HSL), and adenosine monophosphate-activated protein kinase (AMPK). The results showed a significant decline in the serum concentration of total cholesterol, LDL-cholesterol, and triglycerides, although there was a significant increase in serum levels of HDL-cholesterol and glycerol in rats received forskolin alone or with mevastatin when compared with control and dyslipidemic groups. The mRNA expression levels of AC, HSL, and AMPK were significantly increased in omental adipose tissue of rats received forskolin when compared with other groups. In conclusion, forskolin acts synergistically with mevastatin to lower lipid profile and improve lipid metabolism in dyslipidemic rats through upregulation of AMPK expression.

## 1. Introduction

Dyslipidemia is a potent risk factor for the incidence of coronary heart diseases. People, those who keep their lipid profile normal, are usually away from bad consequences of dyslipidemia [1]. On behave of lifestyle interventions, statins are routinely used to lower the risk of dyslipidemia consequences with aspirin; the concept to treatment of dyslipidemia has been widely debated and of heterogeneous aspects [2]. Commonly, it has been known that dyslipidemia is associated with overproduction of many atherogenic lipoproteins as well as it lead to decrease the high density lipoproteins (HDL) and increases low density lipoproteins (LDL) [3]. Many compounds have been approved to be used in treatment or control dyslipidemia; natural agents are widely used for this prospect [4–7]. The target for these agents mainly is to reduce LDL and increase HDL. While statins are used in the same purpose, but their efficiency to reduce LDL is 50-60% [8]. So, seeking for synergetic compounds help statins and maximize their actions on lipid profile, and metabolism is a mandatory object. Here, we can introduce forskolin (FSK) as helper for statins. Forskolin, the new/old compound that extracted from plant *Coleus forskohlii*, is used widely to stimulate adenylate cyclase (AC) that lead to elevate intracellular cAMP levels [9, 10]. So it has been used for controlling many disorders like glaucoma [11], nerve impairments [12], and improve nerve cells [13], and blood pressure [14]. As well as it has been used in many studies for controlling of hyperlipidemias [15, 16]. On the other way, adenosine monophosphate-activated protein kinase (AMPK) is activated when there is a decline in ATP levels inside the mammal cells. It has been recorded that AMPK has a pivotal role in both control and the development of obesity as well as played a great role in lipid metabolism [17]. Mevastatin was isolated from the blue-green mold *Penicillium citrinum*; it had significant structural similarity to HMG-CoA and was a competitive inhibitor of HMG-CoA reductase, with an association constant 10,000-fold higher than HMG-CoA [18]. The study is aimed at examining the synergic action of both mevastatin and forskolin 9 in dyslipidemic rats through their effect on the regulation of genes controlling lipid metabolism and lipid profile.

## 2. Material and Methods

### 2.1. Animals

#### 2.1.1. Animal Selection and Management

Forty males of albino rats with weight 200-250 gm were used in the present study (animals were obtained from the animal house Faculty of Veterinary Medicine, Zagazig University). All animals were housed in stainless cages at 22°C with a 12/12 h light/dark cycle and 50% humidity. All rats were provided a standard rat diet and water *ad libitum* one week before the beginning of the experiment. The Institutional Animal Care and Use Committee at Zagazig University (ZU-IACUC/3/F/29/2018) approved the present study.

#### 2.1.2. Animal Grouping

The animals were divided randomly into four groups of 10 animals in each group as follows: *group A*, rats were received normal diet (standard diet for rats), and they were not expose to any type of treatment and were served as a control group; *group B*, rats that received a single dose of Triton WR 1339 (500 mg/kg), and this group was served as a dyslipidemic group; *group C*, rats that received a single dose of Triton WR 1339 (500 mg/kg) with FSK for successive 4 weeks (this group received FSK after confirmation of dyslipidemia); and *group D*, rats that received a single dose of Triton WR 1339 (500 mg/kg) then received forskolin with mevastatin for 4 weeks and were used to study the synergetic effect of both forskolin and mevastatin on dyslipidemic rats.

#### 2.1.3. Body Weight

The mean body weight has been determined at the end of experimental periods and expressed by grams.

#### 2.1.4. Induction of Dyslipidemia

Dyslipidemia is induced by a single dose (500 mg/kg, i.p) of nonionic detergent Triton WR 1339. Triton WR 1339 is a nonionic detergent isooctylpolyoxyethylene phenol, with common formula (C14H22O.C2H4O.CH2O) [19]. The dyslipidemia is confirmed through the given results in Table 1.

#### 2.1.5. Forskolin Administration

Animals received FSK (100% FSK extract 0.5 mg/kg/day) by gastric tube. The total period for FSK administration was 4 weeks.

#### 2.1.6. Mevastatin Administration

 

### 2.2. Material

#### 2.2.1. Forskolin Extract

*Coleus forkohlii* root extract (100 mg) with forskolin (10 mg) was obtained from iHerb Company (http://www.iherb.com/) with product code APF-00025 and UPC code 896996000250 (Pasadena, California, United States).

#### 2.2.2. Triton WR 1339

Triton WR 1339 with CAS no. 25301-02-4 and molecular formula and weight C17H28O3 and 280.40200, respectively, was purchased from Sigma-Aldrich (Sigma-Aldrich Chemie GmbH, Export Department, Eschenstrasse5, 82024 Taufkirchen, Germany).

#### 2.2.3. Mevastatin

Mevastatin powder with CAS no. 73573-88-3, molecular formula C23H34O5, and weight 390.51 was purchased from Sigma-Aldrich (Sigma-Aldrich Chemie GmbH, Export Department, Eschenstrasse5, 82024 Taufkirchen, Germany).

### 2.3. Sampling

#### 2.3.1. Blood Samples

The blood was collected 24 hours at the end of each experimental period (4 weeks). Animals were sacrificed; then, the blood was collected and centrifuged at 5000 rpm for separation of serum. Sera were stored at -20°C until the biochemical examinations were conducted.

#### 2.3.2. Tissue Samples

Omental adipose tissue was obtained from the rat's abdomen then was washed in saline and stored at -80°C until it is used in the gene expression and molecular biological analysis.

### 2.4. Biochemical Determinations

Serum total lipids, total cholesterol, HDL-cholesterol, LDL-cholesterol, triglycerides, and concentrations were determined according to Cheng et al. [20], Freedman et al. [21], and Ghorbani and Abedinzade [22], respectively. Lipolysis markers, glycerol [23], and free fatty acids were determined in the serum of all experimental animals according to Mannion et al. [24].

### 2.5. Molecular Biological Determinations

The mRNA expression levels of AMPK, adenylate cyclase (AC), hormone-sensitive lipase (HSL) genes, and B-actin were determined in omental adipose tissue using RT-PCR. Total RNA was extracted from omental adipose tissues of all experimental rats at the end of the experimental period using RNeasy Mini Kit for RNA extraction from Qiagen Biotech. The isolated RNA was quantitated by measuring its concentration at absorbance 260 nm, and its purity was checked by the ratio between the absorbance values at 260 and 280 nm ratios of 1.8 or more indicate a pure sample. The samples that have purity less than 1.8 were reextracted again; then, all RNA samples were stored at -80°C. Then, the first strand of DNA was synthesized using Qiagen RT-PCR kits. Primers for AMPK, AC, HSL, and actin are synthesized using (primer 3) online software (http://bioinfo.ut.ee/cgi-bin/primer3-0.4.0/primer3) like the following: AMPK*α*1 (ID: 65248); forward, 5′-ATCCGCAGAGAGATCCAGAA-3′ and reverse 5′-CGTCGACTCTCCTTTTCGTC-3′ [25]; AC (NM_007405), sense 5′-ACATTCTTCCCAAGGACGTG-3′ and antisense 5′-GGTCATGSGTGCTGGCATTT-3′; HSL (NM_012859), sense 5′-AGACACCAGCCAACGGATAC-3′ and antisense 5′-TGTGSTGTTCCCCGAAGGAC-3; and B-actin (V01218), sense 5′-CACGGCATTATCACCAACTG-3′ and antisense 5′-GGCAGAGGATTCAAAAGCTG-3′. Real-time PCR reaction program included was installed for all components except polymerase were heated 5 min at 95°C and cooled to 72°C for the activation of the polymerase. Reactions were cycled 35 times for one minute at 95°C, one minute at 60°C, and three minutes at 72°C followed by a final 10 minutes at 72°C. Then, the number of cycles of threshold (Ct) was detected.

## 3. Statistical Analysis

The data were statistically analyzed by SPSS version 20 statistical packages (IBM, New York, NY, USA). Data were presented as a mean ± SE, *n* = 10. Statistical differences between the groups were determined using a one-way analysis of variance (ANOVA). Duncan's test was used for testing the intergrouping homogeneity. Statistical significance was set at *p* < 0.05.

## 4. Results

### 4.1. Mean Body Weight

The body weight is significantly declined in the groups received forskolin or forskolin with mevastatin when compared with control or dyslipidemic rats. The BWT tended to be increased in dyslipidemic rats (Table 2).

#### 4.1.1. Biochemical Investigations

The possible effect of FSK or FSK with mevastatin administration on the lipid profile is shown in Table 3. There was a significant decline in the serum levels of total lipids, total cholesterol, LDL-cholesterol, and triglycerides if compared with dyslipidemic rats. The synergetic action of forskolin and mevastatin was clear and induce more normalization of lipid profile parameters (Table 3). The serum levels of both glycerol and free fatty acids were increased in the dyslipidemic rats when compared to the control group, while they were still high in the FSK and FSK + mevastatin groups indicating a high degree of lipolysis (Table 4).

#### 4.1.2. Molecular Biological Investigations

**Figure 1:**
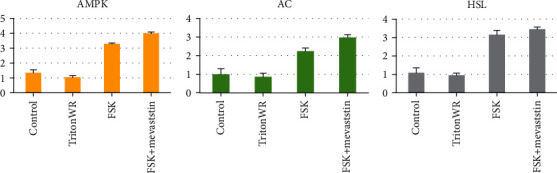
The effect of forskolin on the mRNA expression levels of adenosine monophosphate kinase, adenylate cyclase, and hormone-sensitive lipase genes.

The mRNA expression of adenosine monophosphate kinase (AMPK), adenylate cyclase (AC), hormone-sensitive lipase (HSL), and B-actin is shown in Figure 1; there was a significant increase in the expression of AC and HSL in obese rats treated with forskolin if compared with their control groups.

## 5. Discussion

In the present study, the ability of forskolin (FSK) to neutralize dyslipidemia and increase the lipolysis in omental adipose tissue of dyslipidemic rats was studied. The dyslipidemic rat model was generated using Triton WR 1339. The agent was widely used to study the lipid metabolism status in experimental animals and did not involve inducing great dramatic changes in blood parameters and has minor side effects and is the model of choice when study lipid profile [19]. Serum cholesterol and triglycerides were elevated in the sera of experimental rats after a single dose of Triton WR 1339 (Table 3). Records proved that the normalization usually occurred after 10 days after the administration of treatment agents [19]. The importance of such a study come to introduce a base of elucidation for the ability of FSK to normalize lipid profile and stimulate lipolysis, especially in omental adipose. *Secondly*, it examined the influence of FSK on the molecular level of genes controlling the lipolysis. *Thirdly*, as it is a natural agent, FSK can be used with wide-scale and safe agents for controlling obesity/fat deposition. The study was designed to solve the great bias about the ability of FSK to induce both lipolysis and reduction of serum lipid contents. It does so through its ability to induce visceral fat lipolysis and aid in lipids uptake by cells. So, it seems to be ideal for obese persons. The study based on the biochemical determinations of both serum lipid profile, lipolysis parameters, and molecular investigations of gene expressions of enzymes regulates lipolysis in adipose tissues, AMPK, AC, and HSL. According to our data, FSK seems to be able to decline the body weight of dyslipidemic rats, the matter which intensify its role as a weight controller, and the effect was more obvious when it synergistically used with mevastatin. Biochemically, it appeared to decline serum total lipid level in dyslipidemic rats alone, and its action seemed to be powerful when it administered with mevastatin. The serum total lipid level was decline 1.3- and 1.4-fold in dyslipidemic rats received FSK or FSK with mevastatin (Table 3). The question here is how FSK be able to normalize serum total lipid profile in obese rats although it increases fat lipolysis. According to our results, FSK was able to reduce all malignant circulating lipids in the serum of experimental rats. It declined total cholesterol (1.3- and 1.5-fold), LDL-cholesterol (1.7- and 2.4-fold), and triglycerides (1.25- and 1.22-fold) while it was able to elevate glycerol and free fatty acids (as indicators for adipose tissue lipolysis) (1.3- and 1.6-fold) and HDL-cholesterol (1.5- and 1.6-folds) in dyslipidemic rats received FSK (Table 3). Before answering the abovementioned question, we should observe that the rate by which FSK stimulates lipolysis is nearly equal to the rate by which it reduces serum lipid profile. It was well-known that FSK can stimulate lipolysis through its action to stimulate AC activity and so promote cAMP production [26]. Its ability to do so has been approved even at low concentrations [27]. The ability of FSK to induce both lipolysis and reduction of serum lipid profile is regarding many mechanisms; a long time ago, it has been approved that FSK prevents the conversion of circulating glucose to lipids [28], the matter which will direct all glucose for glycolysis, and so, it will reduce fat storage and do so also through stimulation of insulin secretion [29]. But the most important explanation for the ability of FSK to reduce serum lipid profile is its ability to induce the uptake of lipids by the body cells helping in ideal fat distribution [30, 31]. In the same line, many authors proved that FSK also reduces the contents of fats in the body cells [32, 33], which explain its ability to direct the stored fat to biosynthetic pathways. On the other hand, it was very clear from our results that FSK was able to induce visceral adipose tissue lipolysis; it is approved by the existence of the high level of serum glycerol and free fatty acids in experimental rats shown in Table 4. From its definition, lipolysis is a process of breakdown of triglycerides in adipose tissues into free fatty acids and glycerol [34]. According to this, we have used the glycerol and free fatty acid levels in the serum of experimental rats as indicators of lipolysis process. The results reflect what already happened in adipose tissue; there was a high level of glycerol and FFAs with a reduction of triglyceride levels in the blood of experimental rats that have been received FSK alone or with mevastatin during the experimental period (Table 4). It has been approved that FSK stimulates glycerol and free fatty acids release from animal adipocytes [35], human adipose tissue [36], adipocytes cell lines [37, 38], and mice adipose tissue [39]. Recently, FSK has been used as a new supplement for controlling obesity [40] while it has been found that FSK can decrease total body lipids percentage; this comes with an improvement for lean body mass [41]; this comes in the same line of our results. One of the most important observations in our results was the ability of FSK to reduce blood cholesterol levels; there was a decline in the total LDL-cholesterol and total cholesterol with a significant elevation in HDL-cholesterol concentrations in the serum of obese rats received FSK. The explanation for the ability of FSK to reduce serum cholesterol is due to its ability to promote expression of many genes that stimulate cholesterol uptake through activation of LDL receptor gene expression and activate the biosynthesis of HMG-CoA reductase [42]. It has been found also that FSK has the ability to cooperate with some genes to shuttle cholesterol from outer to inner mitochondrial membrane, the matter with magnifying its role in cholesterol deposition and reducing its levels in serum [30]. On the other hand, FSK has been used to promote the synthesis and production of progesterone; by the way, it leads to increase the uptake of cholesterol by the cells and reducing its levels in serum [31]. Also, FSK restores cholesterol deposition in human macrophages [43]. A triangle of three genes was studied to elucidate the normalization effect of FSK on the lipid profile. AMPK-AC-HSL mRNA expression levels were studied in the omental adipose tissue. The expressional level of all three genes was increased in FSK or FSK+ groups when compared with the dyslipidemic rats' group. Recent records have proved that AMPK is a key enzyme that can modulate lipolysis, lipogenesis, and fatty acid synthesis through phosphorylation of the key substrates [17]. The relationship between AMPK and HSL has been studied. Controversial data have been published regarding this relationship. Many experiments suggested the powerful role of protein kinase PKA to induce phosphorylation of HSL the matter which activates it in adipose tissues; the process itself is very important in its translocation to lipid droplets for induction of its action [44, 45]. While other records proved that AMPK induced HSL inhibition by phosphorylation [46]. Others demonstrated that AMPK can induce lipolysis but through the stimulation of TG hydrolase [47]. In general, AMPK was approved to induce lipolysis under basal conditions never under the stimulated conditions, and even if AMPK inhibited HSL, there was a high free fatty acid release [48]. In our study, both AMPK and HSL expression levels were increased in the FSK treated groups when compared with dyslipidemic ones. Coming in the same line of the records reported that both activity and lipolysis were not inhibited by AMPK and the inhibition activity of AMPK is associated with tissue-specific functions [49]. Another side of our triangle is the relationship between AMPK and AC. Several studies reported that the upregulation of cAMP can affect the AMPK activity been known to be an effective 26,11. Studies also suggested that overexpression of AC can potentiate AMPK activity, the matter which improving the energy balance in the cell [50]. In our model, both FSK induce overexpression of both AC and AMPK in adipose tissue of dyslipidemic rats. FSK stimulated AC activity from a long time ago [51]. The overexpression of AC in groups leading to an increase in the lipolysis in adipose tissue and also the expression levels of AC were increased in groups received FSK with mevastatin; this improves the ability of FSK to protect also against obesity. On the other hand, HSL is an intracellular enzyme able to break a variety of esters and is considered to be affecting fat deposition/release in adipose tissue [52]. Gene expression profile of HSL was analyzed in omental adipose tissues of experimental rats, and the effect of FSK on HSL mRNA expression level and its influence on lipolysis were analyzed by real-time RT-PCR. First of all, it has been known that triglyceride (TG) breakdown was originally begin through the action of HSL, although some studies have improved that deletion of the HSL gene in knockout models did not greatly affect the induction of this lipolysis in adipose tissue; as HSL is mainly specific for diacylglycerol (DAG) breakdown, others improved that HSL knockout mice have shown near-complete deficiency of lipolysis [53]. It has been known that FSK increases PKA activity through the activation of cAMP release; this will lead to phosphorylation of HSL which indeed leads to increase lipolysis of adipose tissue [54]. Our data obtained in the present study showed significantly high expression levels of HSL mRNA in adipose tissue in all rats received FSK. The increase of HSL expression levels leads to an increase in the release of FFAs and glycerol from adipocytes, and this also was observed from our data obtained in the present study. Others reported that inhibition of HSL can lead to the accumulation of diacylglycerols (DAGs) in adipocytes [55], and this is in the same line as our obtained results. It has been revealed that lipolysis in adipose tissue was associated with an increase in HSL expression levels [56]. In the same line of our data, the association of HSL expression levels and its role to induce lipolysis in adipose tissue has been improved in many recent studies. Our results also showed a low expression level of HSL in adipose tissue of dyslipidemic rats; in the same line of our data, authors have improved that HSL mRNA expression level has been decreased in adipose tissue of obese men [57], while it has been increased for 5-fold in adrenocortical cell lines treated with FSK [58]; this comes following our findings. The results of the present study emphasized the synergetic action of both FSK and mevastatin on both lipid profiles and genes in dyslipidemic rats. Data regarding the use of both FSK and mevastatin to study lipid metabolism was widely reported [42]. The discovery of existed statins induces a revolution in the treatment of cholesterol-induced disease due to their role in the inhibition of cholesterol biosynthesis [59]. Although, the use of statin in the treatment itself is associated with many side effects like myalgia [60], serum transaminase elevations, and acceleration of the onset of diabetes mellitus [61]. It is still a vital therapy in patients with dyslipidemia [59]. It has been demonstrated that mevastatin could activate AMPK signaling [62]. Hand by hand with its known activity as a competitive inhibitor to HMG-CoA reductase [63], it leads to reduce cholesterol synthesis. Regarding to the present results, dyslipidemic rats received FSK with mevastatin showed a great reduction in lipid profile parameters with the lowest glycerol level; this illustrated the synergic action of both FSK and mevastatin to normalize lipids in dyslipidemic rats.

## 6. Conclusions

Forskolin synergistically with mevastatin induced normalization of lipid profile and lipolysis of omental lipids in dyslipidemic rats through the overexpression of AMPK, AC, and HSL.

## Figures and Tables

**Table 1:** 

Parameter	Control	Triton WR 1339
Total cholesterol (mg/dL)	109.61 ± 11.5	252.08 ± 11.2
TG (mg/dL)	91.42 ± 10.8	1449.82 ± 101.8

**Table 2:** Effect of forskolin administration on body weight (gm).

Groups	Body weight (gm)
Control	235.2 ± 9.5^c^
Triton WR 1339	320.09 ± 10.2^d^
FSK	201.02 ± 11.1^b^
FSK + mevastatin	199.45 ± 8.2^a^

**Table 3:** Effect of forskolin administration on lipid profile in dyslipidemic rats (mg/dL).

Parameter	Total cholesterol	HDL-c	LDL-c	TG
Control	109.61 ± 11.5^d^	66.37 ± 11^a^	36.64 ± 11.1^d^	91.42 ± 10.8^d^
Triton WR 1339	252.08 ± 11.2^a^	39.34 ± 10.4^d^	154.40 ± 10.8^a^	1449.82 ± 101.8^a^
FSK	159.03 ± 11.1^b^	53.63 ± 10.5^c^	92.23 ± 10.6^b^	125.93 ± 13.7^b^
FSK + mevastatin	124.67 ± 11.2^c^	61.74 ± 10.6^b^	69.07 ± 11.2^c^	96.73 ± 12^c^

**Table 4:** Effect of forskolin on lipolysis parameters in dyslipidemic rats.

Parameter	Glycerol (*μ*mol/L)	Free FAs (nmol/*μ*L)
Control	1.64 ± 0.13^b^	0.52 ± 0.05^b^
Triton WR 1339	3.75 ± 0.15^a^	4.22 ± 0.25^a^
FSK	3.42 ± 0.24^a^	4.15 ± 0.31^a^
FSK + mevastatin	1.87 ± 0.17^b^	4.24 ± 0.18^a^

Means with different letter superscripts are significant at *p* < 0.05.

## Data Availability

All data are included in the manuscript, and there is no other supplementary data.

## References

[1] Kopin L., Lowenstein C. (2017). Dyslipidemia. *Annals of Internal Medicine*.

[2] Anderson T. J., Mancini G. B., Genest J., Grégoire J., Lonn E. M., Hegele R. A. (2015). The new dyslipidemia guidelines: what is the debate?. *The Canadian Journal of Cardiology*.

[3] Iqbal J., Al Qarni A., Hawwari A., Alghanem A. F., Ahmed G. (2018). Metabolic syndrome, dyslipidemia and regulation of lipoprotein metabolism. *Current Diabetes Reviews*.

[4] Bertuccioli A., Moricoli S., Amatori S., Rocchi M. B. L., Vici G., Sisti D. (2020). Berberine and dyslipidemia: different applications and biopharmaceutical formulations without statin-like molecules-a meta-analysis. *Journal of Medicinal Food*.

[5] El-Tantawy W. H., Temraz A. (2019). Natural products for controlling hyperlipidemia: review. *Archives of Physiology and Biochemistry*.

[6] Momtazi-Borojeni A. A., Katsiki N., Pirro M., Banach M., Rasadi K. A., Sahebkar A. (2019). Dietary natural products as emerging lipoprotein (a)-lowering agents. *Journal of Cellular Physiology*.

[7] Talebi S., Bagherniya M., Atkin S. L., Askari G., Orafai H. M., Sahebkar A. (2020). The beneficial effects of nutraceuticals and natural products on small dense LDL levels, LDL particle number and LDL particle size: a clinical review. *Lipids in Health and Disease*.

[8] Lamiquiz-Moneo I., Giné-González J., Alisente S. (2020). Effect of bergamot on lipid profile in humans: a systematic review. *Critical Reviews in Food Science and Nutrition*.

[9] Chauhan S., Parida S., Prakash E. (2020). Hyperlipidemia impairs uterine *β*-adrenergic signaling by reducing cAMP in late pregnant rats. *Reproduction*.

[10] Hao H., Ma X., Chen H. (2020). The cyclic adenosine monophosphate elevating medicine, forskolin, reduces neointimal formation and atherogenesis in mice. *Journal of cellular and molecular medicine*.

[11] Wagh V. D., Patil P. N., Surana S. J., Wagh K. V. (2012). Forskolin: upcoming antiglaucoma molecule. *Journal of Postgraduate Medicine*.

[12] Levi A. D., Burks S. S., Anderson K. D., Dididze M., Khan A., Dietrich W. D. (2016). The use of autologous Schwann cells to supplement sciatic nerve repair with a large gap: first in human experience. *Cell Transplantation*.

[13] Wang G., Ma Z., Cao L. (2017). A Novel Method for Obtaining Highly Enriched Schwann Cell Populations from Mature Monkey Nerves Based on In Vitro Predegeneration. *Molecular medicine reports*.

[14] Rocha M. L., Silva B. R., Lunardi C. N., Ramalho L. N., Bendhack L. M. (2016). Blood pressure variability provokes vascular *β*-adrenoceptor desensitization in rats. *Vascular Pharmacology*.

[15] Hu S., Liu K., Luo H. (2019). Caffeine programs hepatic SIRT1-related cholesterol synthesis and hypercholesterolemia via A2AR/cAMP/PKA pathway in adult male offspring rats. *Toxicology*.

[16] Tian F., Ying H. M., Wang Y. Y., Cheng B. N., Chen J. (2020). MiR-542-5p inhibits hyperglycemia and hyperlipoidemia by targeting FOXO1 in the liver. *Yonsei Medical Journal*.

[17] Wang Q., Liu S., Zhai A., Zhang B., Tian G. (2018). AMPK-mediated regulation of lipid metabolism by phosphorylation. *Biological & Pharmaceutical Bulletin*.

[18] Endo A. (2010). A historical perspective on the discovery of statins. *Proceedings of the Japan Academy, Series B*.

[19] Korolenko T. A., Bgatova N. P., Ovsyukova M. V., Shintyapina A., Vetvicka V. (2020). Hypolipidemic effects of *β*-glucans, mannans, and fucoidans: mechanism of action and their prospects for clinical application. *Molecules*.

[20] Cheng Y. S., Zheng Y., VanderGheynst J. S. (2011). Rapid quantitative analysis of lipids using a colorimetric method in a microplate format. *Lipids*.

[21] Freedman D. S., Khan L. K., Dietz W. H., Srinivasan S. R., Berenson G. S. (2001). Relationship of childhood obesity to coronary heart disease risk factors in adulthood: the Bogalusa Heart Study. *Pediatrics*.

[22] Ghorbani A., Abedinzade M. (2013). Comparison of in vitro and in situ methods for studying lipolysis. *International Scholarly Research Notices*.

[23] Nielsen T. S., Jessen N., Jorgensen J. O., Moller N., Lund S. (2014). Dissecting adipose tissue lipolysis: molecular regulation and implications for metabolic disease. *Journal of Molecular Endocrinology*.

[24] Mannion D. T., Furey A., Kilcawley K. N. (2019). Development and validation of a novel free fatty acid butyl ester gas chromatography method for the determination of free fatty acids in dairy products. *Journal of Agricultural and Food Chemistry*.

[25] McCrimmon R. J., Fan X., Cheng H. (2006). Activation of AMP-activated protein kinase within the ventromedial hypothalamus amplifies counterregulatory hormone responses in rats with defective counterregulation. *Diabetes*.

[26] Doseyici S., Mehmetoglu I., Toker A., Yerlikaya F. H., Erbay E. (2014). The effects of forskolin and rolipram on cAMP, cGMP and free fatty acid levels in diet induced obesity. *Biotechnic & Histochemistry*.

[27] Paschoal D. M., Sudano M. J., Schwarz K. R. (2017). Cell apoptosis and lipid content of *in vitro*-produced, vitrified bovine embryos treated with forskolin. *Theriogenology*.

[28] Mills I., Moreno F. J., Fain J. N. (1984). Forskolin inhibition of glucose metabolism in rat adipocytes independent of adenosine 3',5'-monophosphate accumulation and lipolysis. *Endocrinology*.

[29] Zawalich W. S., Zawalich K. C. (1990). Forskolin-induced desensitization of pancreatic beta-cell insulin secretory responsiveness: possible involvement of impaired information flow in the inositol-lipid cycle. *Endocrinology*.

[30] Daems C., Di-Luoffo M., Paradis E., Tremblay J. J. (2015). MEF2 cooperates with forskolin/cAMP and GATA4 to regulate star gene expression in mouse MA-10 Leydig cells. *Endocrinology*.

[31] Moravek M. B., Shang M., Menon B., Menon K. (2016). HCG-mediated activation of mTORC1 signaling plays a crucial role in steroidogenesis in human granulosa lutein cells. *Endocrine*.

[32] Prates E. G., Alves S. P., Marques C. C. (2013). Fatty acid composition of porcine cumulus oocyte complexes (COC) during maturation: effect of the lipid modulators trans-10, cis-12 conjugated linoleic acid (t10,c12 CLA) and forskolin. *In Vitro Cellular & Developmental Biology. Animal*.

[33] Prates E. G., Marques C. C., Baptista M. C. (2013). Fat area and lipid droplet morphology of porcine oocytes during *in vitro* maturation with trans-10, cis-12 conjugated linoleic acid and forskolin. *Animal*.

[34] Baskaran P., Thyagarajan B. (2017). Measurement of basal and forskolin-stimulated lipolysis in inguinal adipose fat pads.

[35] Rui Y., Tong L., Cheng J., Wang G., Qin L., Wan Z. (2017). Rosmarinic acid suppresses adipogenesis, lipolysis in 3T3-L1 adipocytes, lipopolysaccharide-stimulated tumor necrosis factor-*α* secretion in macrophages, and inflammatory mediators in 3T3-L1 adipocytes. *Food & Nutrition Research*.

[36] Michaud A., Pelletier M., Noel S., Bouchard C., Tchernof A. (2013). Markers of macrophage infiltration and measures of lipolysis in human abdominal adipose tissues. *Obesity*.

[37] Li R., Guan H., Yang K. (2012). Neuropeptide Y potentiates beta-adrenergic stimulation of lipolysis in 3T3-L1 adipocytes. *Regulatory Peptides*.

[38] Zhou D., Samovski D., Okunade A. L., Stahl P. D., Abumrad N. A., Su X. (2012). CD36 level and trafficking are determinants of lipolysis in adipocytes. *The FASEB Journal*.

[39] Benz V., Bloch M., Wardat S. (2012). Sexual dimorphic regulation of body weight dynamics and adipose tissue lipolysis. *PLoS One*.

[40] Rios-Hoyo A., Gutierrez-Salmean G. (2016). New dietary supplements for obesity: what we currently know. *Current Obesity Reports*.

[41] Godard M. P., Johnson B. A., Richmond S. R. (2005). Body composition and hormonal adaptations associated with forskolin consumption in overweight and obese men. *Obesity Research*.

[42] Geng Y., Hsu J. J., Lu J. (2011). Role of cellular cholesterol metabolism in vascular cell calcification. *The Journal of Biological Chemistry*.

[43] Odnoshivkina Y. G., Sytchev V. I., Petrov A. M. (2017). Cholesterol regulates contractility and inotropic response to *β*2-adrenoceptor agonist in the mouse atria: involvement of G i -protein–Akt–NO-pathway. *Journal of Molecular and Cellular Cardiology*.

[44] Anthony N. M., Gaidhu M. P., Ceddia R. B. (2009). Regulation of visceral and subcutaneous adipocyte lipolysis by acute AICAR-induced AMPK activation. *Obesity*.

[45] Yeaman S. J. (2004). Hormone-sensitive lipase--new roles for an old enzyme. *The Biochemical Journal*.

[46] Daval M., Diot-Dupuy F., Bazin R. (2005). Anti-lipolytic action of AMP-activated protein kinase in rodent adipocytes. *The Journal of Biological Chemistry*.

[47] Ahmadian M., Abbott M. J., Tang T. (2011). Desnutrin/ATGL is regulated by AMPK and is required for a brown adipose phenotype. *Cell Metabolism*.

[48] Kim S. J., Tang T., Abbott M., Viscarra J. A., Wang Y., Sul H. S. (2016). AMPK phosphorylates desnutrin/ATGL and hormone-sensitive lipase to regulate lipolysis and fatty acid oxidation within adipose tissue. *Molecular and Cellular Biology*.

[49] Watt M. J., Steinberg G. R., Chan S., Garnham A., Kemp B. E., Febbraio M. A. (2004). Beta-adrenergic stimulation of skeletal muscle HSL can be overridden by AMPK signaling. *The FASEB journal*.

[50] Jayarajan V., Appukuttan A., Aslam M., Reusch P., Regitz-Zagrosek V., Ladilov Y. (2019). Regulation of AMPK activity by type 10 adenylyl cyclase: contribution to the mitochondrial biology, cellular redox and energy homeostasis. *Cellular and Molecular Life Sciences: CMLS*.

[51] Insel P. A., Stengel D., Ferry N., Hanoune J. (1982). Regulation of adenylate cyclase of human platelet membranes by forskolin. *The Journal of Biological Chemistry*.

[52] Fang X., Zhao Z., Jiang P., Yu H., Xiao H., Yang R. (2017). Identification of the bovine HSL gene expression profiles and its association with fatty acid composition and fat deposition traits. *Meat Science*.

[53] Wu J. W., Preuss C., Wang S. P. (2017). Epistatic interaction between the lipase-encoding genes Pnpla2 and Lipe causes liposarcoma in mice. *PLoS Genetics*.

[54] Wakai E., Aritake K., Urade Y., Fujimori K. (2017). Prostaglandin D_2_ enhances lipid accumulation through suppression of lipolysis via DP_2_ (CRTH_2_) receptors in adipocytes. *Biochemical and Biophysical Research Communications*.

[55] Engin A. (2017). Fat cell and fatty acid turnover in obesity. *Advances in Experimental Medicine and Biology*.

[56] Silvério R., Lira F. S., Oyama L. M. (2017). Lipases and lipid droplet-associated protein expression in subcutaneous white adipose tissue of cachectic patients with cancer. *Lipids in Health and Disease*.

[57] Petridou A., Chatzinikolaou A., Avloniti A. (2017). Increased triacylglycerol lipase activity in adipose tissue of lean and obese men during endurance exercise. *The Journal of Clinical Endocrinology and Metabolism*.

[58] Czajkowski M. T., Holysz M., Trzeciak W. H. (2015). Induction of hormone-sensitive lipase/cholesteryl esterase gene expression by C/EBP*α* independently of the PKA pathway in the adrenocortical Y-1 cells. *Steroids*.

[59] Toth P. P., Banach M. (2019). Statins: then and now. *Methodist DeBakey Cardiovascular Journal*.

[60] Banach M., Rizzo M., Toth P. P. (2015). Statin intolerance - an attempt at a unified definition. Position paper from an International Lipid Expert Panel. *Expert opinion on drug safety*.

[61] Preiss D., Seshasai S. R., Welsh P. (2011). Risk of incident diabetes with intensive-dose compared with moderate-dose statin therapy: a meta-analysis. *JAMA*.

[62] Lin Z., Zhang Z., Jiang X. (2017). Mevastatin blockade of autolysosome maturation stimulates LBH589-induced cell death in triple-negative breast cancer cells. *Oncotarget*.

[63] Yao Q., Ma L., Liu L. (2017). Hydroxylation of Compactin (ML-236B) by CYP105D7 (SAV_7469) from *Streptomyces avermitilis*. *Journal of Microbiology and Biotechnology*.

